# Billroth-I anastomosis in distal subtotal gastrectomy for non-early gastric adenocarcinoma

**DOI:** 10.2478/raon-2023-0041

**Published:** 2023-09-04

**Authors:** Sevak S Shahbazyan, Mushegh A Sahakyan, Artak Gabrielyan, Xiaoran Lai, Aram Martirosyan, Hmayak Petrosyan, Shushan Yesayan, Artur M Sahakyan

**Affiliations:** Department of General Surgery, Shengavit Medical Center, Yerevan, Armenia; Department of General & Laparoscopic Surgery, Yerevan State Medical University after M. Heratsi, Yerevan, Armenia; The Intervention Center, Oslo University Hospital, Oslo, Norway; Department of Research & Development, Division of Emergencies and Critical Care, Oslo University Hospital, Oslo, Norway; Department of Surgery N1, Yerevan State Medical University after M. Heratsi, Yerevan, Armenia; Department of General and Abdominal Surgery, ArtMed MRC, Yerevan, Armenia; Oslo Centre for Biostatistics and Epidemiology, University of Oslo, Oslo, Norway; Department of Anesthesiology, ArtMed MRC, Yerevan, Armenia

**Keywords:** gastrectomy, anastomosis, Billroth-I, Billroth-II, adenocarcinoma

## Abstract

**Background:**

Billroth-I (B-I) anastomosis is known as a simple and physiological reconstruction method after distal subtotal gastrectomy for early gastric cancer. Yet its role and oncological validity in non-early gastric adenocarcinoma (NEGA) remain unclear.

**Patients and methods:**

Patients with NEGA without distant metastases operated between May 2004 and December 2020 were included. Surgical and oncologic outcomes of distal subtotal gastrectomy were studied in patients with B-I and Billroth II (B-II) anastomoses. Propensity score matching (PSM) was used to adjust for age, gender, tumor size, location, resection type, pT and pN stages.

**Results:**

A total number of 332 patients underwent distal subtotal gastrectomy for NEGA followed by B-I and B-II anastomoses in 165 (49.7%) and 167 (50.3%) cases, respectively. B-I was applied in patients with smaller tumor size, less advanced pT stage and tumor location in the gastric antrum. The former was also associated with lower proportion of multiorgan resections and shorter operative time. After PSM, these differences became statistically non-significant, except operative time. Postoperative outcomes were similar before and after PSM. Greater lymph node yield was observed in patients with B-I anastomosis. The incidence of recurrence, specifically local recurrence was lower in patients with B-I anastomosis. However, this association was not statistically significant in the multivariable model. Median overall survival was 38 months, without significant differences between the groups.

**Conclusions:**

The use of B-I anastomosis after distal subtotal gastrectomy for NEGA is associated with satisfactory surgical and oncologic outcomes. B-I anastomosis should be considered as a valid reconstruction method in these patients.

## Introduction

The incidence of distal gastric cancer has fallen in the Western countries over the last decade.^1^ However, it still prevails in Asia and other parts of the world.^2^ Stomach body and pyloric antrum are the most common sites for gastric cancer among the Armenian population.

Distal subtotal gastrectomy with adequate lymphadenectomy is the cornerstone in the treatment of resectable distal gastric cancer. After resection, the continuity of gastrointestinal tract can be restored through different reconstruction methods, such as Billroth I (B-I), Billroth II (B-II) and Rouxen-Y. The two latter are based on a closure of the duodenal stump and formation of gastro-jejunal anastomosis, while B-I is performed by creating gastro-duodenal anastomosis. Thus, the main advantages of B-I over B-II and Roux-en-Y are its technical simplicity and retaining the physiological route for food passage. Several comparative studies between the above-mentioned techniques have been published to date.^3,4,5^ In general, these focus on short-term results and/or long-term functional outcomes by examining heterogenous patient cohorts including those with early gastric cancer. While B-I is widely used in surgery for early gastric cancer, its long-term oncologic results in non-early distal gastric adenocarcinoma (NEGA) remain unclear. Furthermore, some concerns have been raised in the literature regarding its oncological safety.^6^

Current study aimed to examine the oncologic safety of performing B-I anastomosis following distal subtotal gastrectomy for NEGA.

## Patients and methods

Patients underwent distal subtotal gastrectomy for gastric adenocarcinoma at Kanaker-Zeytun Medical Center and ArtMed Medical Rehabilitation Center (both in Yerevan, Armenia) between May 2004 and December 2020.

Neoadjuvant chemotherapy was utilized in a negligible number of cases (2.1%). Tumor ingrowth into adjacent major vessels (the celiac axis, the common hepatic artery, the superior mesenteric artery/vein, portal vein) was considered as a contraindication for gastrectomy. All procedures were performed by one surgeon (AMS) via laparotomy. D2 was the standard extent for lymphadenectomy in all patients with NEGA. B-I and B-II anastomoses were used for restoring the continuity of gastrointestinal tract after resection. The choice of reconstruction method was left at surgeon's discretion. The prerequisite for performing B-I was ensuring no tension between the gastric and duodenal stumps. In some cases, the gastric stump was mobilized up until the short gastric vessels to avoid tension. Although the Kocher manoeuvre was not performed routinely, the proximal section of the duodenum was mobilized sufficiently (and cut 1cm distal from the pylorus) to insure negative resection margin. If tumor extended to the upper part of the stomach or was located very close to the pylorus, B-II reconstruction was considered. The latter was performed end-to-side, approximately 40 cm distal to the ligament of Treitz via the anterocolic pathway. B-II was accompanied with Braun anastomosis created 25 cm distal to the gastrojejunostomy.

Patient follow-up included instrumental examinations and evaluation of serum tumor markers 3 and 6 months postoperatively and then every 6 months within the first 5 years after surgery. Chest and abdominal computed tomography were performed 1 year after surgery and then repeated annually.

### Study design

Outcomes of distal subtotal gastrectomy for NEGA were compared between the patients who had received B-I anastomosis and those who had received B-II anastomosis. The primary endpoints of this study were long-term oncologic outcomes, namely, recurrence and survival. Secondary endpoints intra- and postoperative outcomes.

Patient demographics, clinical presentation and perioperative parameters were prospectively registered in the database throughout the study period. The long-term oncologic data were obtained from outpatient hospital visits and telephone interviews. Propensity score matching (PSM) was applied to minimize selection bias. Propensity scores were based on age, gender, tumor location, tumor size, type of resection, tumor stage and nodal stage as we believe most of these factors may influence the choice of anastomosis technique. Patients with either of these variables missing were excluded from the matching procedure.

The study protocol was considered by the accredited Institutional Review Board for Medical Ethics. The requirement for approval was waived by the ethics committee due to the retrospective nature of this study.

All patients who had undergone distal subtotal gastrectomy for NEGA within the study period met the inclusion criteria for this study. NEGA was defined as stage IB-IIIC gastric adenocarcinoma confirmed on final pathology. Patients with NEGA who had undergone surgical procedures other than distal subtotal gastrectomy, were excluded from the analysis. So were those with distant metastases or with gastric tumors other than adenocarcinoma.

### Definitions

Extended gastrectomy was defined as en-bloc resection of adjacent organs and structures due to clinically verified tumor invasion (cT4b stage gastric cancer) as described elsewhere.^7^ Morbidity was defined according to Clavien and Dindo.^8^ Grade ≥ IIIa complications were considered severe.

The 8^th^ edition of American Joint Committee on Cancer (AJCC) staging manual for gastric cancer was used for TNM classification and disease staging.^9^ Tumor size was determined by its morphometric measurement at the pathology work-up. R0 was defined as no microscopic residual cancer at the resection margins.

Tumor recurrence was diagnosed based on radiological evidence of intra-/extra-abdominal soft tissue and/or signs of peritoneal carcinomatosis. Three types of tumor recurrence were reported in this study – local recurrence, distant metastases and peritoneal carcinomatosis. Overall survival was defined as the time between the date of surgery until the date of death from any cause or the date of censoring. Data were censored at the last follow-up.

### Statistics

Data were analysed using R version 4.2.2. Continuous variables are presented as mean (± standard deviation) and median (range) for normally and non-normally distributed data, respectively. The two-sample T-test was used to compare normally distributed data, while the Mann-Whitney *U* test was used for non-normally distributed data. Categorical data are presented as frequencies (percentages). The Chi-square test or Fisher's exact test, when applicable, were applied to compare categorical variables. A two-tailed p-value < 0.05 was considered statistically significant.

PSM was applied to achieve balanced groups with comparable baseline characteristic and potentially minimizing confounding. Logistic regression was performed to estimate the propensity to undergo two different surgical procedures for gastric cancer. The R package ‘MatchIt’ (version 4.5.0) was used to create the final matched cohort. The matching was done using one-to-one nearest neighbour propensity score matching without replacement within a predefined propensity score radius (ie, caliper = 0.1) with a propensity score estimated using logistic regression of the treatment on the covariates. After matching, all standardized mean differences were below 0.1, indicating adequate balance. For the propensity-score matched cohort, paired methods were used in the analysis. The paired T-test was utilized for normally distributed data, and the Wilcoxon signed-rank test was employed for non-normally distributed data. Categorical variables were analysed using McNemar's test.

A multivariable binary logistic regression model with backward selection was used to examine the association between disease recurrence and clinicopathological parameters significant in the univariable analysis (p-value < 0.05). Median survival was estimated by using the Kaplan-Meier method and survival curves were plotted. The log-rank test was used to compare median survival between the groups.

## Results

### Short-term outcomes

A total number of 332 patients underwent distal subtotal gastrectomy for NEGA. B-I and B-II reconstructions were performed in 165 (49.7%) and 167 (50.3%) patients, respectively.

After applying PSM, 97 patients were analyzed in each group. These were comparable in terms of age, gender, and body mass index (Table 1). The total number of comorbidities was greater in patients receiving B-II reconstruction. Before PSM, the use of B-I reconstruction was associated with the smaller tumor size (4.5 *vs*. 6.2 cm, p = 0.001), tumor location in gastric antrum (97% *vs*. 79.6%, p = 0.001) and multi-organ resections were less common in this group (1.8 *vs*. 15%, p = 0.001). These differences became statistically non-significant after matching. Despite PSM, operative time with B-I remained shorter in comparison to B-II (143 *vs*. 165 min, p = 0.001). Other postoperative outcomes were similar in the two matched groups.

**TABLE 1.** Perioperative data in patients with non-early gastric adenocarcinoma undergoing distal subtotal gastrectomy

**Variable**	**Unmatched**			**Propensity score matched**		
	
	**B-I (n = 165)**	**B-II (n = 167)**	**p-value**	**B-I (n = 97)**	**B-II (n = 97)**	**p-value**
Age, years, mean (SD)	60.8 (11.7)	62.6 (10.9)	0.16	60.9 (10.9)	63.2 (11.6)	0.46
Gender (female), n (%)	74 (44.8%)	59 (35.3%)	0.08	42 (43.3%)	43 (44.3%)	0.26
BMI, kg/m^2^, mean (SD)	26.0 (6.5)	25.5 (5.1)	0.55	26.3 (5.9)	25.4 (5.5)	0.31
Comorbidity, n (%)	129 (78.2%)	109 (65.3%)	0.01	76 (78.3%)	63 (64.9%)	0.04
Cardiovascular disease, n (%)	88 (53.3%)	75 (44.9%)	0.13	50 (51.5%)	46 (47.4%)	0.58
Diabetes mellitus, n (%)	18 (10.9%)	16 (9.6%)	0.69	14 (14.4%)	7 (7.2%)	0.13
Number of comorbidities, mean (SD)	2.3 (1.1)	3.1 (0.9)	0.001	2.3 (1.1)	3.0 (0.8)	0.001
ASA score (III–IV), n (%)	145 (87.9%)	145 (86.8%)	0.77	87 (89.7%)	88 (90.7%)	0.81
Hemoglobin, g/dL, mean (SD)	124 (27)	119 (29)	0.09	122 (28)	119 (29)	0.42
Total protein, g/dL, mean (SD)	72 (8.4)	72 (5.9)	0.53	70.5 (9.8)	72.1 (5.8)	0.15
CEA, ng/mL, median (range) [IQR]^*^	1 (0.5–627.2) [1–2]	1.5 (0.5–188) [1–3]	0.02	1 (0.5–174) [1–2]	1 (0.5–100) [1–2]	0.81
Ca 19-9, U/mL, median (range) [IQR]^*^	8 (1–1549) [3–21]	9 (1–999) [3–29]	0.62	8.3 (1–1549) [4–25]	9 (1–999) [3–30]	0.7
Location in antrum, n (%)	160 (97%)	133 (79.6%)	0.001	93 (95.9%)	94 (96.9%)	0.65
Extended gastrectomy, n (%)	3 (1.8%)	25 (15%)	0.001	3 (3.1%)	4 (4.1%)	0.65
Operative time, min, mean (SD)	144 (28)	168 (29)	0.001	143 (27)	165 (28)	0.001
Red blood cell transfusion, n (%)	17 (10.3%)	13 (7.8%)	0.42	12 (12.4%)	7 (7.2%)	0.23
Severe complications, n (%)	8 (4.8%)	13 (7.8%)	0.27	5 (5.2%)	7 (7.2%)	0.53
Anastomotic leakage, n (%)	4 (2.4%)	5 (3%)	1.0	1 (1%)	2 (2.1%)	0.56
Relaparotomy, n (%)	7 (4.2%)	11 (6.6%)	0.35	4 (4.1%)	5 (5.2%)	0.71
30-day mortality, n (%)	1 (0.6%)	2 (1.2%)	1.0	1 (1%)	2 (2.1%)	0.56
90-day mortality, n (%)	1 (0.6%)	4 (2.4%)	0.37	1 (1%)	4 (4.1%)	0.36
Postoperative days, median (range)	11 (6–48) [9–13]	11 (5–72) [9–13]	0.51	10 (6–48) [9–13]	11 (5–72) [9–14]	0.47

ASA = American Society of Anesthesiologists; B-I = Billroth I; B-II = Billroth II; BMI = body mass index; CA 19-9 = carbohydrate antigen 19-9; CEA = *carcinoembryonic antigen*; IQR = *interquartile range; SD = standard deviation;*

* = data missing in 36 patients

The two matched groups were not significantly different in terms of tumor size pT, pN, AJCC stages, R0 resection rates and tumor differentiation (Table 2). Significantly greater mean lymph node yield was observed in patients who had received B-I anastomosis (27 *vs*. 19, p = 0.001). Other lymph node-related parameters such as total number of positive lymph nodes and lymph node ratio were comparable between the two methods.

**TABLE 2.** Pathology findings in patients with non-early gastric adenocarcinoma undergoing distal subtotal gastrectomy

**Variable**	**Unmatched**			**Propensity score matched**		
	
	**B-I (n = 165)**	**B-II (n = 167)**	**p-value**	**B-I (n = 97)**	**B-II (n = 97)**	**p-value**
Tumor size, cm, mean (SD)	4.5 (1.7)	6.2 (2.4)	0.001	5.1 (1.5)	5.2 (1.5)	0.52
pT stage, n (%)			0.046			0.28
T1–T2	69 (41.8%)	49 (29.3%)		32 (33%)	31 (32%)	
T3	70 (42.4%)	85 (50.9%)		44 (45.4%)	53 (54.6%)	
T4a/T4b	26 (15.8%)	33 (19.8%)		21 (21.6%)	13 (13.4%)	
pN stage, n (%)			0.089			0.62
N0	47 (28.5%)	55 (32.9%)		27 (27.8%)	32 (33%)	
N1	31 (18.8%)	17 (10.2%)		14 (14.4%)	10 (10.3%)	
N2	38 (23%)	34 (20.4%)		25 (25.8%)	23 (23.7%)	
N3a	32 (19.4%)	47 (28.1%)		19 (19.6%)	24 (24.7%)	
N3b	17 (10.3%)	14 (8.4%)		12 (12.4%)	8 (8.2%)	
Disease stage (AJCC), n (%)			0.1			0.95
I B	29 (17.6%)	26 (15.6%)		13 (13.4%)	15 (15.5%)	
II A	32 (19.4%)	26 (15.6%)		17 (17.5%)	18 (18.6%)	
II B	29 (17.6%)	16 (9.6%)		16 (16.5%)	14 (14.4%)	
III A	33 (20%)	36 (21.6%)		24 (24.7%)	21 (21.6%)	
III B	25 (15.2%)	41 (24.6%)		15 (15.5%)	20 (20.6%)	
III C	17 (10.3%)	22 (13.2%)		12 (12.4%)	9 (9.3%)	
Detected lymph nodes, mean (SD)	27 (12)	18 (9)	0.001	27 (12)	19 (10)	0.001
Positive lymph nodes, mean (SD)	6 (7)	6 (7)	0.98	6.4 (8.1)	5.6 (7.2)	0.44
Lymph node ratio, mean (SD)	0.21 (0.25)	0.28 (0.27)	0.52	0.24 (0.27)	0.28 (0.27)	0.27
R0 resection, n (%)	151 (91.5%)	120 (71.9%)	0.001	87 (89.7%)	80 (82.5%)	0.16
Tumor differentiation, n (%)			0.018			0.51
Well	30 (18.2%)	22 (13.2%)		15 (15.5%)	15 (15.5%)	
Middle	44 (26.7%)	60 (35.9%)		26 (26.8%)	33 (34%)	
Poor/non-differentiated	91 (55.1%)	85 (50.9%)		56 (57.7%)	49 (50.5%)	

ASA = American Society of Anesthesiologists; B-I = Billroth I; B-II = Billroth II; BMI = body mass index; *SD = standard deviation;*

* = data missing in 36 patients

### Long-term outcomes

**FIGURE 1.**
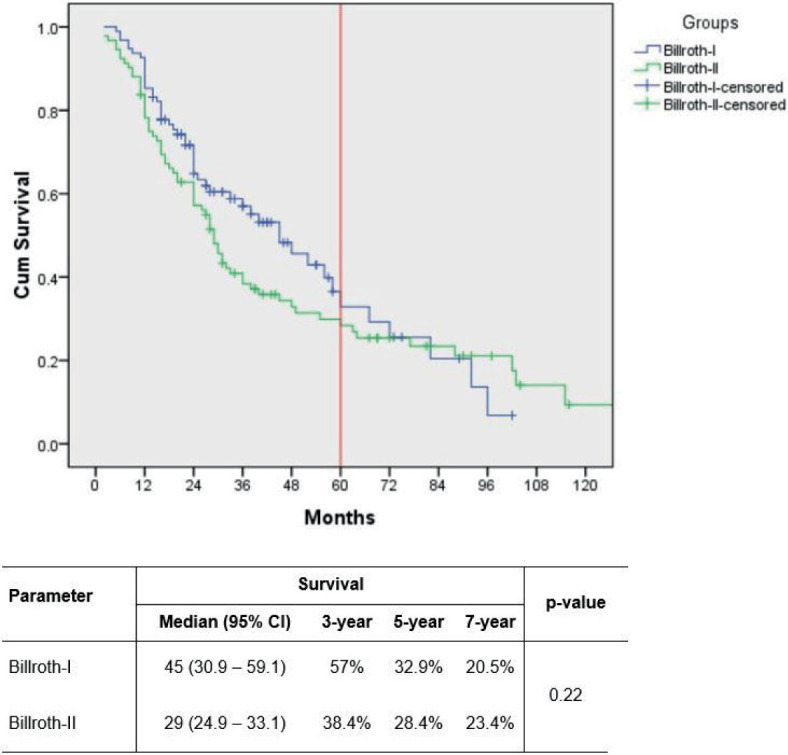
Survival in patients with non-early non-metastatic gastric cancer undergoing subtotal distal gastrectomy with Billroth I and Billroth II reconstruction – propensity-matched analysis (1:1).

Median follow-up for the patients was 28 (2–150) months. Less than one-third (29%) of the patients received adjuvant chemotherapy overall and no significant differences were found between the groups (Table 3). The recurrence rate was lower following B-I in the unmatched cohort. The difference became statistically non-significant after applying PSM – 21.9% *vs.* 35.8%, p = 0.054. When analyzing for specific types of recurrence, local recurrence was significantly lower in patients with B-I reconstruction (3.1% *vs.* 11.6%, p = 0.022). No significant differences were found for other types of recurrence. Median survival was 38 (30–46) months, while 3- and 5-year survival rates were 52 and 32%, respectively. Overall survival was not significantly different between the groups after PSM (Figure 1).

**TABLE 3.** Long-term oncologic outcomes after distal subtotal gastrectomy with Billroth I vs Billroth II reconstruction for non-early gastric adenocarcinoma^*^

**Variable**	**Unmatched**			**Propensity score matched**		
	
	**B-I (n = 164)**	**B-II (n = 165)**	**p-value**	**B-I (n = 96)**	**B-II (n = 95)**	**p-value**
Adjuvant chemotherapy, n (%)^¶^	52 (31.7%)	44 (26.7%)	0.32	36 (37.5%)	26 (27.4%)	0.18
Recurrence, n (%)^¶^	34 (20.7%)	60 (36.4%)	0.002	21 (21.9%)	34 (35.8%)	0.054
Recurrence type, n (%)^¶^						
Local	4 (2.4%)	16 (9.7%)	0.006	3 (3.1%)	11 (11.6%)	0.022
Distant metastases	12 (7.3%)	25 (15.2%)	0.025	8 (8.3%)	12 (12.6%)	0.3
Peritoneal carcinomatosis	18 (11%)	19 (11.5%)	0.88	10 (10.4%)	11 (11.6%)	0.65
Overall survival, months, median	45 (35.4–54.6)	29 (24.3–33.7)	0.036	45 (30.9–59.1)	29 (24.9–33.1)	0.22
3-year	59%	41.1%		57%	38.4%	
5-year	34.6%	28.7%		32.9%	28.4%	

* = Patients who died after surgery (n = 3) were excluded from the analysis of long-term oncologic outcomes;

¶ = data not available in 3 patients

Uni- and multivariable analyses were performed to identify prognostic factors for tumor recurrence following distal subtotal gastrectomy for NEGA (Table 4). According to the univariable analysis, operative time, reconstruction method, pT and pN stages, total number of harvested lymph nodes, lymph node ratio and R1 resection were associated with recurrence. In the multivariable analysis, reconstruction method was not associated with recurrence. pT stage and lymph node ratio were the only independent predictors for tumor recurrence.

**TABLE 4.** Uni- and multivariable analysis of prognostic factors for tumor recurrence following distal subtotal gastrectomy for non-early gastric adenocarcinoma (Propensity score matching [PSM] cohort)

**Variable**	**Univariable**		**Multivariable**	
	
	**Odds ratio (95% CI)**	**p-value**	**Odds ratio (95% CI)**	**p-value**
Age, years	0.99 (0.96–1.01)	0.33		
Gender (female)	1.34 (0.71–2.51)	0.37		
BMI, kg/m^2^	0.99 (0.93–1.07)	0.89		
Comorbidity	0.74 (0.38–1.46)	0.39		
ASA score (III–IV)	0.86 (0.31–2.4)	0.78		
CEA, ng/mL	1.0 (0.99–1.02)	0.59		
Ca 19-9, U/mL	1.0 (0.99–1.001)	0.93		
Extended gastrectomy	0.99 (0.19–5.26)	0.99		
B-II reconstruction (*vs.* B-I)	1.99 (1.05–3.78)	0.035	————————————	—
Operative time	1.012 (1.001–1.024)	0.038	————————————	—
Estimated blood loss	1.001 (0.99–1.01)	0.65		
Red blood cell transfusion	0.26 (0.06–1.18)	0.082		
Severe complications	2.06 (0.53–7.96)	0.29		
Relaparotomy	2.59 (0.62–10.74)	0.19		
Tumor size	1.13 (0.92–1.38)	0.25		
pT stage				
T1–T2	reference		reference	
T3	3.86 (1.76–8.48)	0.001	3.13 (1.36–7.19)	0.007
T4a/T4b	1.04 (0.32–3.35)	0.95	0.64 (0.14–2.86)	0.56
pN stage				
N0	Reference		————————————	—
N1	1.82 (0.57–5.82)	0.32	————————————	—
N2	2.31 (0.89–5.95)	0.08	————————————	—
N3a	4.31 (1.69–10.94)	0.002	————————————	—
N3b	3.18 (0.98–10.26)	0.05	————————————	—
Detected lymph nodes	0.96 (0.94–0.99)	0.022	0.96 (0.93–1.01)	0.06
Lymph node ratio	1.05 (1.01–1.09)	0.025	1.01 (1.0–1.03)	0.043
R0 resection	3.2 (1.36–7.55)	0.008	2.41 (0.85–6.84)	0.1
Tumor differentiation				
Well	reference			
Middle	1.18 (0.59–2.37)	0.65		
Poor	0.63 (0.25–1.59)	0.33		
Adjuvant chemotherapy	1.28 (0.66–2.47)	0.46		

ASA = American Society of Anesthesiologists; B-I = Billroth I; B-II = Billroth II; BMI = body mass inde, CA 19-9 = carbohydrate antigen 19-9; CEA = *carcinoembryonic antigen*

## Discussion

Our findings suggest that B-I anastomosis is a valid option in patients undergoing distal subtotal gastrectomy for NEGA. Therefore, B-I should be considered in these patients when it is technically feasible and does not compromise the oncologic outcomes. At the same time, factors such as tumor extent, its proximity to the pylorus and the size of the gastric stump should be considered when deciding on reconstruction method.

The results from this study demonstrate that oncologic outcomes after distal subtotal gastrectomy with B-I anastomosis are comparable to those after distal subtotal gastrectomy with B-II. Surprisingly though, the number of detected lymph nodes in patients with B-I anastomosis was higher compared with those with B-II. At the same time, B-I was mostly applied in the later period (2013–2020), while B-II was predominantly applied in the earlier period (2004–2012). Most likely, this represents changes in meticulousness of pathology work-up as surgical technique and the extent of lymphadenectomy did not change at our institution over time. Since the proportion of B-I was greater in the later period, we assume it has inadvertently coincided with an improved pathology work-up resulting in greater lymph node yield in this group.

Patients with B-I reconstruction were found to have lower incidence of recurrence compared to those with B-II. However, this correlation did not remain statistically significant in the PSM cohort and multivariable analysis. Low incidence of local recurrence in the B-I group demonstrates that B-I technique is oncologically justified in patients with NEGA undergoing distal subtotal gastrectomy. On the contrary, a higher rate of local recurrence after distal subtotal gastrectomy with B-II anastomosis may indicate the need for a more radical approach (ie, total gastrectomy) in some of these patients. In other words, one can assume that preserving proximal stomach in these patients to obtain better quality of life after surgery was probably not always oncologically justified.

Another important implication of this study is that it comes from a developing country with high incidence of gastric cancer, where no screening programs are utilized. As a result, most of the patients undergoing surgery for distal gastric cancer are diagnosed with NEGA. This is in contrast with the situation in the Asian countries, where about 50% of patients present with early gastric cancer.^10,12^ While gastric disfunction and health-related quality of life issues are important endpoints after surgery for early gastric cancer, survival and oncologic results are central in gastrectomy for NEGA.

There are several limitations in this report worth mentioning. First and foremost, this is a retrospective study with its inherent biases. Second, no strict criteria were in place when opting for B-I or B-II anastomosis after distal subtotal gastrectomy. Thus, it was left at surgeon's discretion. Third, since this study was based on single-surgeon experience, the generalizability of our findings is limited.

B-I was associated with postoperative outcomes that were not inferior to those of B-II. Furthermore, the long-term oncologic results are not compromised when using B-I. Hence, the latter can be considered as an appropriate reconstruction method in patients with NEGA that are candidates for distal subtotal gastrectomy.

## Conclusions

The use of B-I anastomosis after distal subtotal gastrectomy for NEGA is associated with satisfactory surgical and oncologic outcomes. B-I anastomosis should be considered as a valid reconstruction method in these patients.
